# Music Evolution in the Laboratory: Cultural Transmission Meets Neurophysiology

**DOI:** 10.3389/fnins.2018.00246

**Published:** 2018-04-16

**Authors:** Massimo Lumaca, Andrea Ravignani, Giosuè Baggio

**Affiliations:** ^1^Center for Music in the Brain, Department of Clinical Medicine, Aarhus University and The Royal Academy of Music Aarhus/Aalborg, Aarhus, Denmark; ^2^Artificial Intelligence Lab, Vrije Universiteit Brussel, Brussels, Belgium; ^3^Research Department, Sealcentre Pieterburen, Pieterburen, Netherlands; ^4^Language and Cognition Department, Max Planck Institute for Psycholinguistics, Nijmegen, Netherlands; ^5^Language Acquisition and Language Processing Lab, Department of Language and Literature, Norwegian University of Science and Technology, Trondheim, Norway

**Keywords:** cultural transmission, diffusion chains, signaling games, iterated learning, music universals, music diversity, neural predictors, mismatch negativity (MMN)

## Abstract

In recent years, there has been renewed interest in the biological and cultural evolution of music, and specifically in the role played by perceptual and cognitive factors in shaping core features of musical systems, such as melody, harmony, and rhythm. One proposal originates in the language sciences. It holds that aspects of musical systems evolve by adapting gradually, in the course of successive generations, to the structural and functional characteristics of the sensory and memory systems of learners and “users” of music. This hypothesis has found initial support in laboratory experiments on music transmission. In this article, we first review some of the most important theoretical and empirical contributions to the field of music evolution. Next, we identify a major current limitation of these studies, i.e., the lack of direct *neural* support for the hypothesis of cognitive adaptation. Finally, we discuss a recent experiment in which this issue was addressed by using event-related potentials (ERPs). We suggest that the introduction of neurophysiology in cultural transmission research may provide novel insights on the micro-evolutionary origins of forms of variation observed in cultural systems.

## Introduction

There has recently been a surge of interest in the biological and cultural origins, and evolution of music (Wallin et al., 2001; McDermott and Hauser, 2005; Patel, 2010). Music is prominent in virtually all human societies, and in its most sophisticated versions it is only attested in humans. This fact raises two important questions: how did music originate? And how did it evolve in its current forms? One intriguing issue here, especially in relation to the cognitive and neural bases of music evolution (Honing et al., 2015), is that of the *evolution of musical structure*. Musical systems are structured at several levels, from melody and harmony to rhythm and composition, in ways that may resemble the organization of other human generative systems, such as language (Jackendoff and Lerdahl, 1983; Jackendoff, 2009). The analogy between language and music may be pushed further, if one considers aspects of music that may be understood “semantically.” Listening to music can evoke a wide range of extra-musical experiences, from emotional feelings (e.g., the sadness suggested by Albinoni's Adagio in G minor) to the mental imagery of specific referents (e.g., characters or ideas in Wagnerian *Leitmotifs*) (Patel, 2010). Musical structures can and often do relate to a world of possible experiences and non-musical phenomena (Lerdahl, 2003) *expressively* (by being associated to internal affective states, e.g., emotional qualities), if not *representationally* (via relations of reference and truth, as language does) (Patel, 2010).

In this work, we focus on the cultural origins of musical syntax: the set of principles governing the combination of melody and rhythm into “well-formed” sequences (for a discussion on the evolution of semantic structures see Lumaca and Baggio, 2017, 2018; Ravignani and Verhoef, 2018). Some aspects of musical syntax, such as the organization of temporal structure and pitch intervals, display widespread distribution and striking cross-cultural similarities. For example, the tendency to use small intervals in non-polyphonic melodic phrases, or “proximity,” has been observed across several musical traditions of the world, including indigenous tunes from North America, Europe, and Asia (Dowling, 1968; Von Hippel, 2000). Despite some exceptions, such as Scandinavian and Swiss yodeling music, proximity is a prominent feature of melodic structure. These shared attributes are known as “musical universals.” Nevertheless, their form and frequency differ across and within different musical traditions of the world (Lomax, 1977; Rzeszutek et al., 2012; Savage et al., 2015). How can we explain both the invariance and the variation of structure in music? Which processes underlie the cross-cultural convergence toward common music traits or their diversification? In this paper, we suggest that neuroscience can provide critical methodological and theoretical tools for testing and generating hypotheses on this complex matter.

This article is organized as follows. We start by presenting a recent theoretical perspective in which music is understood as an evolving cultural system, adapting to the human brain [sections Linking Biological and Cultural Levels of Analysis and From Cultural Transmission to Neurophysiology (and Back)]. In section The Cognitive Level: Diffusion Chains and the Evolution of Musical Regularities in the Lab, we describe studies that support this view using data from behavioral experiments. In section The Neural Level: Constraints Imposed by a Neuronal Niche Drive the Emergence of Regularities, we transpose our analysis of cultural adaptation to the neural level. Partly using the “neuronal recycling hypothesis” as a theoretical framework (Dehaene and Cohen, 2007), we argue that music can adapt to a “neuronal niche” defined by the specific information processing constraints imposed by neural circuits originally evolved for auditory streaming.

To our knowledge, no one until recently has investigated this hypothesis by means of brain imaging or neurophysiology. In section Neural Predictors in Cultural Evolution Research, we describe a recent experiment in which this hypothesis was tested combining behavioral and neurophysiological methods. Finally (section The Neural Origins of Cultural Variation), we suggest that the introduction of concepts and methods from neuroscience in music evolution, and cultural evolution in general, can provide new insights on the process of cultural variation.

### Linking biological and cultural levels of analysis

Music may be seen as a complex adaptive system, shaped by various biological, environmental, and cultural factors. This has made it difficult for musicologists and cognitive scientists to analyze the evolutionary origins of musical structure. The predominant view during the last century was the *cultural account*, where music was seen as an entirely socio-cultural construct, free to vary with virtually no biological and environmental constraints on its structure and content (Nettl, 1983; Repp, 1991; Blacking et al., 1995). The striking diversity of musical forms, as attested across and within cultures, and over human history, seems to support this notion (Lomax, 1968; Henry, 1976). Yet, this account has been challenged by experiments in psychology and neuroscience, together supporting a broadly *biological account* of the origins of music. Several studies point to the existence of perceptuo-cognitive biases and constraints in music processing and production (e.g., Trehub, 2000; Drake and Bertrand, 2001; Zatorre, 2001; Peretz and Zatorre, 2005; Deutsch, 2012) with some parallels in other species (Fitch, 2015). On this view, prototypical properties of music, such as a relatively steady beat, smooth melodic contours, tonality, and a narrow distance between adjacent tones (or “pitch proximity”), derive from built-in functional properties of the brain (McDermott and Hauser, 2005), which tend to manifest themselves in most human cultures (Lerdahl, 1992; Savage et al., 2015).

A recent view is that neither the “cultural account” nor the “biological account” can independently provide a satisfactory theory of the origins and evolution of musical structure (Trainor, 2015). Cultural accounts typically focus on the evolution of *musical systems*, while biological accounts investigate the evolution of the *human capacity to perceive, appreciate, and produce music* (also including musicality; Honing et al., 2015). These different accounts, however, may be connected within a more complete explanatory framework, if one accepts that music is neither an entirely arbitrary cultural construct nor strictly a biological product. Much like natural language, music is a cultural construct, which nonetheless rests upon, and is partly shaped by, human neurobiology. Our neurobiological makeup determines the scope and constraints of human auditory memory capacity, hierarchical sequence processing, attention, perceptual hearing threshold, and auditory scene analysis (Snyder, 2008; Deutsch, 2012). This is now a central tenet in the field of music cognition, and it is becoming increasingly accepted in cultural analyses of music, too. The open question is *how* neurobiological capacities, biases and constraints manifest themselves in actual musical systems (Trainor, 2015).

### From cultural transmission to neurophysiology (and back)

Answering this question requires theories, models, and empirical data that can effectively bridge the gap between the classical chasms of (cultural) evolutionary science: between individual-level and population-level processes, micro-evolutionary and macro-evolutionary processes (Mesoudi, 2011). Specifically, one important question is how the individual's neurobiological endowment manifests itself in music at the population level. This issue was already known in linguistics as the “problem of linkage” (Kirby, 1999). A possible answer is “through cultural transmission.” Music, much like language, is not only a richly structured symbolic system, but also a set of behaviors that is maintained over time by intergenerational transmission (Morley, 2013; Le Bomin et al., 2016).

During intergenerational transmission, cultural information must survive a “memory bottleneck” (Deacon, 1997): the set of all neurobiological biases or constraints that bind our capacity to infer (and store) the “rules” that govern a system of information^1^. The properties of the cultural system that fit best the human neurobiological filter—e.g., those that make information easier to process, encode, and recall—will have greater likelihood of being passed on to the next generation. If this view is correct, in the long run the neurobiological endowment of individuals should be reflected in the musical corpus at the population-level.

This view of transmission, emphasizing *adaptation of fast-changing cultural systems to a largely stable neurocognitive architecture*, was developed in evolutionary linguistics to account for the emergence of structure in human languages, including putative linguistic universals (Christiansen and Chater, 2008). Recent methodological advances (Mesoudi, 2015; Edmiston et al., 2018) have provided support for this view in controlled laboratory conditions. In most experiments, groups of individuals engage in simple, controlled forms of knowledge transmission, for example from a participant (a sender) to another (a receiver), along a diffusion chain. Each participant represents a “generation,” and each interaction between participants allows for the passage of information across generations (Esper, 1925; Bartlett, 1932). The set of items transmitted along a diffusion chain (e.g., linguistic or musical phrases) is a finite sample drawn from the (infinite) set of items that learners have to generalize from. A challenge for research on cultural transmission is to show that core properties of the artificial systems being transmitted are also properties of the actual cultural systems being modeled and that the mechanisms at work in artificial conditions are also at work in real cultural evolution. In a landmark study, Kirby et al. (2008) showed how miniature “languages” emerge in the course of transmission from initial random associations of signals and meanings. When these pairings are transmitted across “generations” of participants, some regularities emerge, including *compositionality* (Hockett, 1960), as observed in human language. This result supports the view that core properties of language can be explained by the interplay of individual cognitive biases (sensu Brighton et al., 2005) and iterated cultural learning and transmission. Recent studies on animal models of cultural learning further support this conclusion (e.g., for non-human primates see Claidière et al., 2014; for a seminal study on zebra finches see Fehér et al., 2009).

One way to start bridging this gap in the musical domain, is to assume that music, like language, is a complex adaptive cultural system, shaped for thousands of years by cycles of transmission, acquisition, and use (Morley, 2013). Following this view, neurobiological biases and constraints, as discussed above, brought out through cultural transmission, would exert effects on the form and structure of music (Merker et al., 2015; Trainor, 2015; Mehr et al., 2018). This mechanism could explain some properties of temporal (rhythm, meter) and spectral (melody, harmony) dimensions of musical structure, which are likely to be the result of *adaptations* to the combined pressures of neural constraints and various socio-cultural forces (Merker, 2006; Merker et al., 2015; Trainor, 2015). This would in principle apply to both *invariants*—putative cultural *universals* shared by musical systems or traditions (Savage et al., 2015)—and *variation* among individuals, generations, and traditions.

This point is not new. Lévi-Strauss (1960) had already observed that some structural regularities observed across cultures (e.g., the fact that symbolic material tends to be organized in binary oppositions) are reflections of principles of brain organization. Therefore, neuroscience is expected to contribute to explanations of the emergence and evolution of structural regularities, including their convergence and diversity. However, to date this issue has been addressed only by behavioral studies, and only to explain some invariant aspects of musical structure. In the next section, we summarize three of these lines of experimental work in the field of music evolution.

## The cognitive level: diffusion chains and the evolution of musical regularities in the lab

In recent experiments, a diffusion chain method was used to study how music evolves in the lab (Ravignani et al., 2016). This study aimed to test whether human psychological biases, amplified by cultural transmission, can explain the emergence of rhythmic universals (Trehub, 2015). In this experiment, participants were given a drumstick and an electronic drum pad. Participants in the first generation listened to 32 randomly generated, hence a-rhythmic, patterns of beats (the input), and were asked to reproduce each of them to the best of their abilities (the output). The “imperfect” output produced by this first generation of participants became the input for the next generation, whose task was to perform the rhythm they heard, and so on, along a diffusion chain. This paradigm is known as “iterated learning” (IL) (Kirby et al., 2008). Given the difficulty to memorize these patterns, errors were introduced in the emerging system of drumming sequences, slightly modifying the original patterns at each generation. Across generations, patterns became increasingly structured and easier to learn. After 8 generations, at the end of each diffusion chain, patterns showed regularities similar to those found across musical traditions of the world. These universal rhythmic regularities included a tendency toward small integer ratios (e.g., 1:1 and 2:1) of intervals between beat durations, and a relatively steady beat, also termed “isochrony” (Savage et al., 2015). This study represents the very first attempt to “grow” musical universals in the lab (Fitch, 2017), and sheds light on the cognitive and cultural mechanisms underlying the creation and vertical transmission of music (Le Bomin et al., 2016).

An IL study by Verhoef (2012) investigated the cultural evolution of combinatorial structures in musical systems. Participants were first exposed to a set of 12 whistles that they had to imitate immediately after listening by using a slide whistle (training phase). Next, they were asked to reproduce the whole set of signals as they remembered it (recall phase). The sequences generated by a participant were used to train the next one in the diffusion chain, and so on, until the end of the chain. In the course of transmission, structural regularities emerged, as predicted by previous computer simulations (de Boer, 2000). In the last generations, fewer discrete units were reused by individuals in concatenations, repetitions, or mirror forms to produce the entire vocabulary of whistles. Combinatoriality is a “design feature” of human language (Hockett, 1960) and it applies to musical structure, too. For instance, the authors observed that two distinct whistles were often combined into a single pattern by the next generation of individuals. Also, participants tended to produce mirror forms out of single patterns, so that more elements were shared between signals of the same set. With fewer units to memorize, organized in this manner, the set of signals was more structured, more compressed, and easier to learn and reproduce.

A more recent attempt to study music evolution in the lab is the work by Lumaca and Baggio (2017). The authors used a different model of cultural transmission than IL: multi-generational signaling games (MGSGs) (Moreno and Baggio, 2015; Nowak and Baggio, 2016). MGSGs are in essence an iterated variant of signaling games (Lewis, 1969; Skyrms, 2010) that combine basic aspects of semiotic models of coordination and communication (e.g., horizontal transmission; Galantucci and Garrod, 2011) with the intergenerational transmission of IL (Kirby et al., 2008). Two-person signaling games were organized in diffusion chains of 8 generations each. In each game, the sender and receiver were expected to converge, through repeated interactions, on a common code: a signaling system where 5 isochronous melodic riffs were associated to basic or compound emotions. This design can contribute to model different aspects of music transmission: first, a degree of alignment of internal states between musical senders (e.g. composers) and receivers (e.g., an audience) at two main levels, the *structural* and *affective* (Temperley, 2004; Bharucha et al., 2011); second, a partial asymmetry in information flow from senders to receivers, which is present in language and music transmission (e.g., from composers to listeners, from teachers to pupils, etc.). In each signaling trial, the sender was presented on the screen with one of the 5 equiprobable emotions (visualized as human facial expressions) and was asked to compose a 5-note isochronous riff on the computer keyboard. The receiver, after he listened to the riff via headphones, was asked to choose one of the 5 expressive faces displayed on the screen (i.e., the one possibly seen by the sender). A feedback was then presented simultaneously to both participants' screens, showing the expressive face seen by the sender and the one chosen by the receiver for the same melodic signal. This procedure was repeated at each successive trial. At the end of the game, the receiver (generation *n*) became the sender in the next game, with the same structure and a new participant as a receiver (generation *n* + 1), and so on, until the chain was completed. Senders were always asked to transmit the code they had learned in the previous game. Therefore, recall errors in the melodic signals (possibly “innovations”) were introduced throughout the experiment. The authors observed the gradual evolution over generations of several structural features of musical phrases: pitch proximity and continuity, symmetry, and motivic structure.

Despite differences in their assumptions and methods, those three experiments have reached similar conclusions: the immediate effects of psychological constraints on the musical systems may be *weak*, but they are *amplified* in the course of inter-generational transmission (Boyd and Richerson, 1988; Kalish et al., 2007; Kirby et al., 2007; Thompson et al., 2016) or iterated reproduction (Jacoby and McDermott, 2017), leading the evolution of musical structures along non-random paths. If principles of auditory organization and memory constraints operate in similar ways also in the production and perception of actual music, they could similarly shape the evolution of historical systems in the course of iterated transmission. Convergence toward some of the musical structures found across populations (Savage et al., 2015) could be then explained, to some extent, by adaptation to a special niche, constituted by a restricted set of low-level perceptual and memory processes. In the rest of the paper we will refer to this special niche as “neuronal niche” (Dehaene and Cohen, 2007).

## The neural level: constraints imposed by a neuronal niche drive the emergence of regularities

In recent years, there has been an increasing interest in how the brain accommodates and shapes novel cultural symbolic systems (Dehaene and Cohen, 2007). A leading hypothesis is that some cortical circuits, initially evolved as a result of specific selective pressures, are later “recycled” to accommodate novel cultural functions (Dehaene and Cohen, 2007; Simon et al., 2013; Dehaene et al., 2015; Skeide et al., 2017). Therefore, the acquisition of novel functions is constrained, however weakly, by prior human evolution. Once “culturally recycled,” pre-existing systems and mechanisms maintain some of their original capacities and limitations, providing a neuronal niche within which culture may adapt and evolve. This also means that the variability observed in cultural systems is limited by brain structure and function across individuals and groups.

If this hypothesis is correct, near-universal characteristics of music (Savage et al., 2015) may be traced back to the computational infrastructure of human auditory cortex and other (e.g., motor, attentional etc.) areas of the brain. Trainor (2015) related the origins of certain invariant musical features as adaptations to bottom-up neural mechanisms of auditory scene analysis (ASA), such as the sequential sound segregation and integration of within-stream elements (Bregman, 1994). These specific mechanisms have evolved specifically to detect and localize multiple sources of auditory objects and to extract regularities from the acoustic environment. They often involve the perceptual grouping of single-event auditory stimuli into auditory streams and operate following Gestalt principles of proximity, similarity, and continuity (Deutsch, 1999). They are automatic (pre-attentive), they emerge early in human development (Demany, 1982; Winkler et al., 2003), and they are widely conserved across species (Fay, 2008). This point shows that the ASA neural circuitry is likely phylogenetically older than human music. Thus, the exaptation (or evolutionary re-use) (Gould and Vrba, 1982) of this more ancient biological mechanism by music should impose constraints on the way music is stored and organized in the brain, and accordingly, on the way it is recalled during transmission. In this regard, perceptual and memory recall advantages have been reported for tone streams that conform to Gestalt principles of organization (Bendixen et al., 2010; Loui, 2012; Rohrmeier and Cross, 2013). The cross-cultural tendency to organize music following these principles (Huron, 2001), in addition to the findings reported by cultural transmission research (Verhoef, 2012; Ravignani et al., 2016; Lumaca and Baggio, 2017), may support the idea that the neurocomputational constraints of the human auditory system constitute a filter through which musical material must pass, adapt, and eventually evolve.

It is surprising that up until recently, no one has attempted to find (counter-) evidence of cultural adaptation using neural measures. Research has shown that even recently-encoded information is shaped by perceptual or memory constraints into more compressed and abstract forms (Tamariz, 2017). Yet, the neural mechanisms underlying this phenomenon remain unknown. One reason is arguably our limited understanding of how information is represented in the brain (Mesoudi et al., 2006). Current whole-brain methods, such as functional magnetic resonance (fMRI), are not well-suited to investigate the precise basis of mental representations (but see Haynes and Rees, 2006; Johnson and Johnson, 2014; Zadbood et al., 2017). Another issue is to establish a link between neural constraints on learning—neural activity underlying specific, fast, and accurate encoding processes (Sadtler et al., 2014)—and cultural adaptation. Electrophysiological methods, such as multi-unit recordings, seem ideal for this purpose, but they are too invasive to be performed on healthy individuals. Various animal models of social learning—in songbirds, primates, and other species—have provided useful information in this respect (Araki et al., 2016; Gadagkar et al., 2016; Tchernichovski and Lipkind, 2016). None of these species possesses cultural behaviors as rich and complex as human music. However, some of their behaviors exhibit structured patterns, which are maintained over time through inter-generational transmission. Cultural transmission, in turn, can shape animal vocal behavior so as to fit species-specific learning constraints (Fehér et al., 2009; Fitch, 2009).

The application of techniques and models used in language evolution allow researchers of animal behavior to explore the biology of culturally transmitted systems in simpler and more controlled conditions, and to answer questions about cultural adaptation that cannot be *directly* answered in humans using current methods (but see next section for *indirect* answers). For example, Araki et al. (2016) used cellular recordings to demonstrate the existence in zebra finches of constraints on neuronal temporal coding that limit song acquisition to certain species-specific temporal features. Juvenile birds acquire their songs by imitating adult tutors. Although zebra finches are not bound to learn only specific sequences, they do show significant consistencies in their vocal repertoires (Lachlan et al., 2016). Do these consistencies result from adaptation of song material to the zebra finch neural constraints on learning? Araki et al. (2016) found that a subset of neurons in the zebra finch auditory cortex responds synchronously and selectively to patterns of inter-syllable silent gap durations, which are typical of their songs. The same cell population was unresponsive to other species' songs. Temporal coding mechanism like this are thought to preserve the species-specific song identity from any random drifts that may be introduced during cultural transmission.

Critically, the same mechanisms might underlie learning behaviors that resemble cultural adaptation in humans. When presented with the songs of other species, zebra finches tend to gradually adjust the duration of inter-syllable intervals toward their own (species-specific) songs' temporal structures, in a way similar to the human adjustment of random auditory stimuli toward Gestalt features. To our knowledge, this work provides the first cellular-level support of the idea of a neurobiological basis of cultural adaptation. It remains to be determined to what extent their findings can be generalized to other species. Would similar neuronal constraints operate in humans? Could they explain perceptual predispositions for some musical features (e.g., for small intervals and isochronous beat)? Are those neuronal constraints species-specific or, instead, are they shared with other species (Nicolai et al., 2014)? Another critical question is whether inter-individual variability in the neural filter is reflected in forms of cultural variation, for example in participant behavior during transmission, or in the shape taken by cultural systems as a result of it. Cross-individual variability is typically regarded as a source of noise in cultural transmission research, and is often removed by means of various procedures. The idea of linking individual neural variability with cultural variation may lend itself well to investigations using brain imaging and electrophysiology, but no one until recently has adopted this approach in cultural transmission research.

### Neural predictors in cultural evolution research

In a recent experiment, Lumaca and Baggio (2016) addressed some of these issues using a neural predictors approach (Berkman and Falk, 2013). This entails use of neuroimaging (fMRI, PET) or electrophysiological methods (EEG/ERPs, MEG) to identify neural predictors of behavior (for examples in the music domain, see Golestani et al., 2002; Zatorre et al., 2012; Zatorre, 2013). Lumaca and Baggio (2016) used neural predictors of signaling behavior as a first approach to examine whether and how symbolic systems adapt to human neural information processing systems, and to assess the effects of inter-individual variation in neural information processing on three core cultural behaviors: social learning, transmission, and regularization of signal sequences. To this purpose, the authors used one of the best-investigated brain signatures of auditory processing, the mismatch negativity (MMN) (Näätänen et al., 1978).

The MMN is a fronto-central negative wave, evoked by violations of some perceptual regularity (Paavilainen, 2013) which is picked up by the brain in a visual or auditory stimulus stream. The limited influence of attentive processes on the MNN (Paavilainen, 2013) and its onset (~200 ms from the relevant stimulus) suggest that the MMN is a *low-level* marker of auditory processing. The encoding of regularities from an auditory input, possibly through the same ASA mechanisms reported above, is an antecedent condition for the elicitation of the MMN (Näätänen et al., 2001). The efficiency of these mechanisms is revealed by the MMN latency and amplitude (Näätänen et al., 1993; Tervaniemi et al., 2001). Larger amplitudes or shorter latencies are typically associated to more accurate representations of the input material and, thus, they are taken as proxies of more efficient encoding mechanisms. The MMN has been used to study how efficiently an individual's auditory system extracts and encodes regularities from acoustic inputs, and how this process may affect linguistic and musical behaviors. For example, differences in ERP responses in infants have been successfully used in various studies to predict cognitive and linguistic development (Molfese and Molfese, 1997; Choudhury and Benasich, 2011). Overall, these studies open up the possibility of using low-level neural markers to predict individual behavior during transmission and acquisition of language, music, and cultural material more generally. Structural properties of symbolic systems may thus be understood as adaptations to information processing bottlenecks during cultural transmission (Kirby, 2001; Tamariz and Kirby, 2015). It should then be possible, for example, to find a relationship between individual brain processing capabilities or limitations, and the degree of regularization imposed by each individual on the cultural material that is being transmitted and acquired.

Neurophysiological (ERP) evidence for this type of effect was provided by Lumaca and Baggio (2016) in the domain of melodic structure. The authors combined ERPs with diffusion chains on two successive days. On day 1, they identified a neural correlate of extracting regularities from 5-tone sequences in musically naïve individuals in a classical auditory oddball paradigm. ERPs were recorded while participants were presented with randomly interleaved standard (80%) and deviant (20%) stimuli: there was no task for the participants, who were watching a silent movie throughout the session. On day 2, participants played a reduced version of MGSGs, with melodic systems of the same kind used by Lumaca and Baggio (2017). Each participant played the first signaling game as receiver (learner) and the second as sender (transmitter)^2^. The main question addressed by the authors was whether constraints and biases on auditory processing could drive the melodic material toward known Gestalt principles of perceptual organization (Lumaca and Baggio, 2017). The results showed that inter-individual variation in neural information processing, as revealed by the latency of the MMN on day 1, predicted learning and transmission of melodic signaling systems in the MGSGs on day 2. Specifically, individuals with longer MMN latencies performed “worse” in the MGSGs, showing lower coordination, transmission, and accuracy. Yet, these participants introduced more innovations than participants with shorter MMN latencies. Inter-individual variation in neural auditory processing (or regularity encoding) may be sufficient to discriminate “better” from “worse” transmitters, as observed in the cultural transmission of music (Sawa, 2002). However, perhaps the most interesting finding was that participants with longer MMN latencies introduced more regularities in the artificial tone system, reproducing more often melodic structures that were more compressed (signals from the same set became more similar), more proximal (temporally adjacent elements in the signals were closer in pitch), and smoother (the sequences showed a coherent melodic direction) than the sequences they originally received. To our knowledge, this study is the first demonstration that three essential processes underlying cultural evolution (i.e., social learning, transmission, and innovation), and three near-universal properties of melodic structure (i.e., proximity, continuity, and compression) are constrained by the organization of sensory and memory systems in the brain. The MMN is only “the tip of the iceberg” here. The MMN is likely to reflect auditory scene analysis and encoding mechanisms. Constraints on these mechanisms, as revealed (among others) by MMN latencies, may represent a “neuronal niche” through which cultural material must pass, adapt, and evolve (see below). In a cultural evolutionary context, this finding may provide clues to the origins of forms of variation observed in cultural symbolic systems. We discuss this point in the next paragraph.

### The neural origins of cultural variation

Human cultural traits show a myriad different forms across world cultures. Music, like language, provides an excellent example of this diversity, within and between populations (Lomax, 1959; Rzeszutek et al., 2012). For instance, the tendency toward the use of intervals of small size or the division of the octave (2:1) into a limited number of tones (or “discreteness”) as observed in several cultures (Merriam et al., 1956; Dowling, 1968) is counterbalanced by significant diversity, within and between those cultures, in the relative frequency of such traits (Savage et al., 2015). The frequency distribution of proximal intervals (<700 cents; Savage et al., 2015) differs across musical traditions, with variation being mostly confined to the interval range 0 (unison) to 6 semitones (Huron, 2001). A similar diversity was found in the “tonal material” of musical cultures (i.e., the total set of discrete pitches within an octave), which spans from the 12 semitones of the Western musical scale to the 22–24 microtonal steps of North Indian and Arabic scales (Malm, 1967; Ayari and McAdams, 2003).

The evolutionary mechanisms that affect the relative frequency of musical characters, such as random cultural drifts and biased selection, have been extensively studied in recent years (Mesoudi, 2015). For example, MacCallum et al. (2012) used a biologically-inspired evolutionary system to explore the effects of “aesthetic” selection on the frequency distribution of musical characters. A population of listeners was asked to rate the pleasantness of randomly generated tunes. The top-rated tunes recombined or mutated into novel variants that were in turn evaluated by a new generation of consumers. The authors reported an over-time increase of characters classically regarded as “musical,” such as isochrony and chordal clarity. This work was the first of its kind to show that consumers' preferences can deeply shape the evolution of music in the near absence of learning and memory pressures. It is still controversial whether aesthetic preferences are just a social construct, changing over time, or if instead they are themselves stable information processing biases (for an in-depth discussion on this topic see Hodges, 2009; Huron, 2009). In a recent model, Reber et al. (2004) combined the two proposals. Specifically, the authors put forward the hypothesis that aesthetic preferences result from an interaction between knowledge-dependent stylistic rules and information processing fluency for certain stimulus properties (e.g., symmetry, clarity, and the amount of information content) (Nieminen et al., 2011). This may explain the evolution of music toward specific features, such as symmetry and chordal clarity (MacCallum et al., 2012; Verhoef, 2012; Lumaca and Baggio, 2017). A similar proposal was made by Haiman (2011) to explain the emergence of symmetric compounds in language. These arguments are still hypothetical, but we are now starting to understand the *effects of these biases* on the cultural evolution of music (Savage and Brown, 2007). Specifically, we know that these processes can enhance the diversity of musical behaviors and forms, but they can also produce local homogeneity^3^. While those mechanisms can explain how musical variants spread over time in a population, the sources of variability remain to a large extent elusive.

Up until now, only four main mechanisms of variation have been considered in music: *creative innovation* (e.g., via original musical composition), *borrowing* (through blending or syncretism), *translation* (from one tonal system to another; Alekseyev, 1986), and *random mutation* (errors in music copying or performance) (Savage and Brown, 2007). Lumaca and Baggio (2016) provided evidence for an additional mechanism: *individual neural variability*. One could argue that every individual in a population represents a distinct and unique “neuronal niche” (Dehaene and Cohen, 2007), through which cultural material is filtered and to which it may eventually adapt. Minor inter-individual differences in neural information processing can manifest themselves in differences in musical behavior. Moreover, they can be amplified and spread via different cultural evolutionary mechanisms. Small differences in learning or information processing can have large system-level effects, if they are amplified by cultural transmission.

One tenet of cultural transmission research is that cultural systems evolve toward certain prior distributions, known as “cognitive attractors” or “inductive biases” (Sperber, 1996; Griffiths et al., 2008). Strong versions of this account have been challenged by recent modeling work (Navarro et al., 2017). The convergence toward priors holds in the (implausible) scenario where all learners are endowed with the same identical prior. However, when learners instantiate (slightly) different constraints, the emerging cultural systems may reflect the more idiosyncratic biases of *some* individuals. In light of our findings, one could suggest that individuals with “tighter bottlenecks” exert a disproportionately large effect on the evolution of musical structures (see Ravignani et al., 2018 for some issues concerning this view). Similarly, differences between populations in brain function and anatomy may, at least in part, be reflected in differences in the structure of the symbolic systems in use. This account has recently found some support in language evolution research. Dediu and Ladd (2007) have shown that the population-level frequencies of two human genes involved in brain growth, Microcephalin and ASPM, are reliably associated with the presence or absence of linguistic tones in that population. The authors' proposal is that variants of these genes may determine small biases at the individual level in the processing and acquisition of linguistic tones, which may in turn give rise to distinct language variants. Those variants are hardly detectable in individual subjects, because tonal and non-tonal languages can be acquired by any individual, independently of genetic variants (Ladd et al., 2008). But when their effects are amplified by inter-generational transmission (Kirby et al., 2008), these variants may give rise to measurable, large-scale population differences.

Dediu and Ladd (2007) is the first study suggesting that variation, as observed in cultural traits and in their distribution, may originate in interindividual neurogenetic variability. Lumaca and Baggio (2016) provide converging neurophysiological evidence in support of this view (for the genetic bases of inter-individual variation in musicality, see Gingras et al., 2015). Genetic and neural variability are not the only source of cultural variation, but they are likely to play a prominent role in any future theory of the biological roots of culture. For example, Brown et al. (2014) have shown that musical and genetic diversity may correlate to some degree. After sampling a set of traditional songs from 9 indigenous populations in Taiwan, they measured the relative distance for 41 properties of song structure and performance-style. Music and genetic distance among the populations were significantly correlated. A similar relation was found in Eurasian populations (Pamjav et al., 2012). The study of genetic and neural variability may help address questions that were considered taboo in ethnomusicology since fairly recently: for example, whether a causal relationship exists between the distribution of some gene variants and aspects of musical systems and behaviors (Jordania, 2006, p. 101; Nikolsky, 2015). Such a theory requires the synergic and coordinated effort of genetics, neuroscience, and research on cultural evolution. The recent drive toward a “grand synthesis” of the latter discipline (Brewer et al., 2017) makes this possibility somewhat more likely.

## Conclusions

In this paper, we have argued that some of the most fundamental (and still unresolved) issues in music evolution can be addressed using the methods of cognitive neuroscience. This approach so far suggests a novel hypothesis on the mechanisms behind forms of cultural variation in musical systems. This line of work can also shed light on the “problem of linkage” (Kirby, 1999). Up until recently, this problem has been framed at only two levels of explanation. At the *behavioral level*, individual behaviors (e.g., code changes) that serve coordination and communication are linked to population-level patterns. At the *cognitive level*, sensory or memory constraints in individuals are identified in order to account for properties (e.g., structural features) of cultural systems. We suggest that a third level, the *neural level*, should be taken into consideration when developing accounts of the origins and evolution of structure in cultural systems, as is the case for accounts of the organization and function of information processing systems (Marr, 1982; Baggio et al., 2012, 2014, 2016). Thus, we can address questions in the cultural domain such as: which sources produce cultural diversity (computational level); through which mechanisms it may arise (e.g., inter-individual variation; algorithmic level); and which physical substrates, if any, those mechanisms exploit (i.e., the human brain; implementational level). We believe that explanations at all three levels are necessary to understand human cultural transmission. This requires (1) analyzing the structural and dynamic properties of the cultural systems (or codes) themselves, (2) determining how those are shaped by perceptual and cognitive biases and constraints, and (3) identifying the biological roots of such biases and constraints using neural and genetic data. This proposal generates several new questions, such as: to what extent do neural processes drive cultural evolution? How does inter-individual variation in brain function and structure affect variation in cultural behaviors? How does the distribution of neural traits in a population affect the structure of the symbolic system itself? How do these traits interact with aesthetic processing biases and the environment at large in the cultural evolution of music? How specific and accurate can neuroprediction get in the context of cultural evolution? Here, we hope to have shown that these questions are worth asking, and are largely amenable to scientific inquiry.

## Author contributions

ML wrote the article. AR and GB made additional contributions and edited the manuscript. All authors approved the manuscript for publication.

### Conflict of interest statement

The authors declare that the research was conducted in the absence of any commercial or financial relationships that could be construed as a potential conflict of interest.

## Footnotes

^1^Our definition of “memory bottleneck” includes constraints on perceptual grouping; capacity and temporal limits of auditory memory, serial processing, and attention; constraints on the neurodynamics of the auditory system; perceptual hearing thresholds. We limited this list to constraints “directly” related to basic aspects of perception and cognition. We acknowledge that constraints of a different nature might have a formative power over musical structures (e.g., motoric, motoric-expressive, physiological, cross-modal, and semantics).

^2^In signaling games with fixed roles, including all MGSGs, the receiver tends to learn the code transmitted by the sender. In other words, there is asymmetry in the division of coordination labor between the sender and the receiver, with most coordination work (most code changes) falling to the latter (Nowak and Baggio, 2016).

^3^The re-use of Wagner's musical ideas by other composers during Nazi Germany and the emergence and maintenance of stylistic clusters in contemporary pop music are clear examples of biased selection.
